# Mitochondrial DNA Haplogroup D4a Is a Marker for Extreme Longevity in Japan

**DOI:** 10.1371/journal.pone.0002421

**Published:** 2008-06-11

**Authors:** Erhan Bilal, Raul Rabadan, Gabriela Alexe, Noriyuki Fuku, Hitomi Ueno, Yutaka Nishigaki, Yasunori Fujita, Masafumi Ito, Yasumichi Arai, Nobuyoshi Hirose, Andrei Ruckenstein, Gyan Bhanot, Masashi Tanaka

**Affiliations:** 1 BioMaPS Institute, Rutgers University, Piscataway, New Jersey, United States of America; 2 Simons Center for Systems Biology, Institute for Advanced Study, Princeton, New Jersey, United States of America; 3 Computational Biology and Bioinformatics, The Broad Institute of MIT and Harvard, Cambridge, Massachusetts, United States of America; 4 Department of Genomics for Longevity and Health, Tokyo Metropolitan Institute of Gerontology, Itabashi-ku, Tokyo, Japan; 5 Department of Longevity and Aging Research, Gifu International Institute of Biotechnology, Kakamigahara, Gifu, Japan; 6 Department of Geriatric Medicine, Keio University School of Medicine, Shinjuku-ku, Tokyo, Japan; 7 Department of Physics, Boston University, Boston, Massachusetts, United States of America; 8 Department of Biomedical Engineering, Rutgers University, Piscataway, New Jersey, United States of America; Innsbruck Medical University, Austria

## Abstract

We report results from the analysis of complete mitochondrial DNA (mtDNA) sequences from 112 Japanese semi-supercentenarians (aged above 105 years) combined with previously published data from 96 patients in each of three non-disease phenotypes: centenarians (99–105 years of age), healthy non-obese males, obese young males and four disease phenotypes, diabetics with and without angiopathy, and Alzheimer's and Parkinson's disease patients. We analyze the correlation between mitochondrial polymorphisms and the longevity phenotype using two different methods. We first use an exhaustive algorithm to identify all maximal patterns of polymorphisms shared by at least five individuals and define a significance score for enrichment of the patterns in each phenotype relative to healthy normals. Our study confirms the correlations observed in a previous study showing enrichment of a hierarchy of haplogroups in the D clade for longevity. For the extreme longevity phenotype we see a single statistically significant signal: a progressive enrichment of certain “beneficial” patterns in centenarians and semi-supercentenarians in the D4a haplogroup. We then use Principal Component Spectral Analysis of the SNP-SNP Covariance Matrix to compare the measured eigenvalues to a Null distribution of eigenvalues on Gaussian datasets to determine whether the correlations in the data (due to longevity) arises from some property of the mutations themselves or whether they are due to population structure. The conclusion is that the correlations are entirely due to population structure (phylogenetic tree). We find no signal for a functional mtDNA SNP correlated with longevity. The fact that the correlations are from the population structure suggests that hitch-hiking on autosomal events is a possible explanation for the observed correlations.

## Introduction

Modern humans have a much higher life expectancy than our closest relatives, the chimpanzees [1]. Amongst humans, the Japanese are amongst the longest lived, with life expectancy of 79 years for males and 86 for females [2]. Whereas this longevity may arise from non-genetic factors such as better health care, diet, lifestyle, etc., it might also have a genetic component, particularly in very old people, who survive to longevity in spite of the lack of any evolutionary selection pressure (except perhaps the so called ‘grandmother effect [3]). In this paper, we focus on mtDNA mutations and analyze whether there is any evidence for their role in longevity. If mtDNA mutations affecting longevity exist, they may either directly confer a selective advantage, or, they may compensate for deleterious mutations on other loci (associated with mtDNA or nuclear genes) or be detrimental (confer a selective disadvantage). Such polymorphisms are not limited to non-synonymous mutations alone. A synonymous mutation on mtDNA may also be a marker for longevity by hitch-hiking, where the mutation is a surrogate for amplification of a haplogroup due to a selective sweep from an autosomal event [4].

Ideally, genetic analysis should include SNPs on both mtDNA and autosomal chromosomes. This would allow a genome wide analysis to identify mutations and pathways associated with longevity and their correlation with geographic clustering and migration histories of population groups as inferred from mtDNA and Y chromosome phylogeny. Unfortunately, although some initial steps have been taken in this direction [5], such detailed data does not yet exist. For the moment, our analysis must be limited to mtDNA sequences.

In this paper we develop techniques to identify mutations for longevity by looking for correlations between the longevity phenotype, mtSNPs and mtDNA haplogroups. The correlations could be due to functional polymorphisms or to population structure. We analyze the correlations using two different methods. In the first, we use several statistical scores to directly test correlations between haplogroups and the longevity phenotype. In the second, we apply Principal Component Spectral Analysis to separate correlations due to functional polymorphisms from those due to population structure. The results are similar. We find that there is a strong selection for Longevity in the D super-haplogroup and strong progressive correlations in the D→D4→D4a lineage. However, using Gaussian generated SNPs distributions we find that this association is most likely due to population structure (ie. it depends on the details of the phylogeny of the M Clade within which D is a sub-clade and does not identify functional SNPs). We identify the mtSNP 14979C (Cytb: Ile78Thr) in D4a to be correlated with extreme longevity. To study whether this mtSNPs might be hitchhiking on nuclear SNPs, we looked for association signatures of enrichment in the Japanese D clade samples in HapMaP [5]. However, the SNP density in HapMaP is too low to allow any definite conclusions.

## Materials and Methods

**Figure 1:**
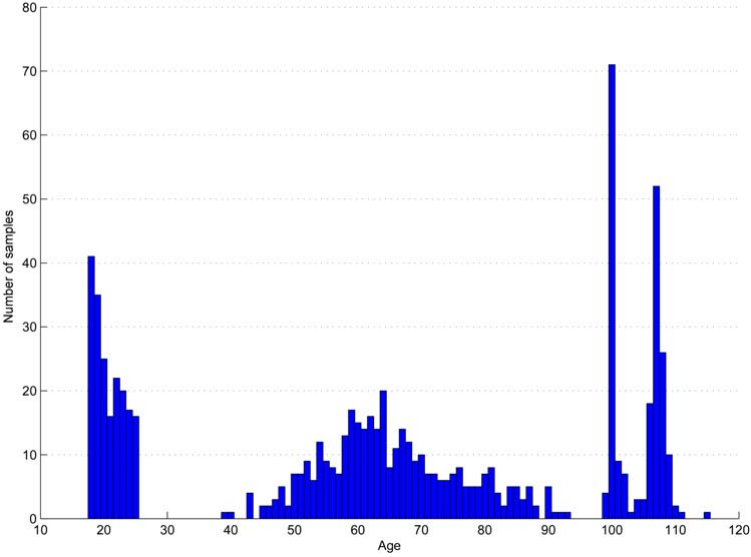
Age distribution across the samples (JD, ND, HN, KA, PD, TC/GC, SSC).

**Figure 1** shows the age distribution of the samples used in the present study. The data comes from two sources. The first is Tanaka et al [6] and consists of 672 complete mitochondrial DNA sequences divided into sets of 96 samples in each of seven phenotypes: JD or diabetic patients with severe angiopathy (mean age: 65±10 years; range 43–92 years), ND or type-2 diabetes mellitus patients (mean age: 58±5 years; range 43–65 years), HN or healthy non-obese young males (mean age: 20±3 years; range 18–25 years), ON or obese young males (mean age: 21±2 years; range 18–25 years), KA or patients with Alzheimer's disease (mean age: 77±10 years; range 47–93 years), PD or patients with Parkinson's disease (mean age: 62±9 years; range 39–81 years) and GC/TC or Gifu/Tokyo centenarians (mean age: 100±1, range 99–105 years) (see [7] and http://mtsnp.tmig.or.jp/mtsnp/index_e.shtml for more information). In addition, we include new data from 112 complete mtDNA sequences from semi-supercentenarians (labeled SSC in this paper), ranging in age from 105–115 years (mean age 107.3±1.2 years). These semi-supercentenarians included 96 females and 16 males and were recruited from all over Japan. The study protocol complied with the Declaration of Helsinki and was approved by the Committees on the Ethics of Human Research of Keio University School of Medicine, Gifu International Institute of Biotechnology, and Tokyo Metropolitan Institute of Gerontology. Written informed consent was obtained from each participant or a member of his/her immediate family. A detailed description of the methods used in the collection, extraction and sequencing of the mtDNA samples used in the present study is given in our website (http://mtsnp.tmig.or.jp/mtsnp/annex_e.html). In brief, sequences were analyzed with a 3130xl Genetic Analyzer from Applied Biosystems. Complete sequences were aligned, assembled, and compared with the program Sequencher 4.1 (Gene Codes, Ann Arbor, MI). For verification, visual inspection of each candidate mtSNP was carried out. At least 2 overlapping DNA templates amplified with different primer pairs were used for identification of each mtSNP. Newly obtained mtDNA sequences of semi-supercentenarians were deposited in GenBank as the following accession numbers (AP010661-AP010772 for SCsq0113-SCsq0113). The 112 new sequences are attached in FASTA format in **Data S1**. The 672 other sequences used in this paper can be downloaded from http://mtsnp.tmig.or.jp/mtsnp/search_mtDNA_sequence_e.html.

The 672 sequences from [6] and the 112 new semi-supercentenarian sequences were pairwise aligned relative to the revised Cambridge Reference Sequence rCRS (http://www.mitomap.org/mitoseq.html) using the Miller-Meyers [8] algorithm as implemented in the “Stretcher” module in EMBOSS, 3H3Hhttp://emboss.sourceforge.net/). We applied a robust technique developed in [9] to the 672 sequences which identified all robust mutations in the data using Principal Component Spectral Analysis [10], [18]–[21] to identify the mutations that represent most of the variation in the data. Following this, we separated the samples into haplogroup clusters by applying an ensemble consensus clustering technique which averages over probabilistic, agglomerative and hierarchical clustering methods and many data perturbations to build an agreement matrix from which a robust set of clusters is determined along with patterns of discriminatory mutations [9]. The optimum number of clusters (kmax) was estimated using the silhouette score [11] and gap-statistics [12]. The robustness and statistical significance of the clusters and mutations was validated by averaging over ten 2:1 training/test bootstrapped datasets on which we repeated the complete clustering analysis. Only 90% or higher sample assignments into clusters and predictive mutations were accepted. This analysis clustered the 672 Japanese samples into 31 robust haplogroups after the samples which had ambiguous membership in the clustering analysis because of small sample size were discarded. The 31 haplogroups, as defined also in Tanaka et al. [6], and confirmed by our unsupervised consensus ensemble clustering technique [9] were: A, B4a, B4b, B4c, B5, D4a, D4b1a, D4b1b, D4b2a, D4b2b, D4d1a, D4d1b, D4e1, D4e2, D4g, D4h, D5a, D5b, F1, F2, M10, M7a, M7b, M8, M9, C, G1, G2, Z, N9a, N9b. We also found 261 SNPs in the patterns of mutation that are characteristic markers for these haplogroups. The 112 semi-supercentenarian sequences were classified into haplogroups based on the patterns of these 261 characteristic SNPs

Out of the 261 mtSNPs identified above, we restricted the analysis to the 235 which were more than 1% polymorphic across the 112 semi-supercentenarian samples. We also eliminated several SNPs in the region from locus 303 to 317 and from 16180 to 16195, which are in the so called hypervariable regions HV1 (16024–16383) and HV2 (57–372). This was because we determined, by examination of the sequences in these regions, that they were length polymorphisms and not true mutations. This left us with a dataset with 227 SNPs and 782 samples which were used for further analysis (see **Table S1** for the complete data used in this paper). The complete previous sequences used in this study (672 samples from [6]) can be downloaded from (http://mtsnp.tmig.or.jp/mtsnp/search_mtDNA_sequence_e.html). The new complete mtDNA sequences of 112 semi-supercentenarians are in **Data S1.**

To measure the degree of association of an mtSNP to longevity, we define a mtSNP enrichment score *L_S_* follows:
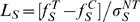



This score is defined for each SNP separately. Consider the case where we are computing this score for centenarians. f_S_^T^ is the fraction of samples in the centenarians that have the SNP, f_S_^C^ is the fraction of samples in the control group (healthy non-obese males) that have the SNP. σ_S_^NT^ is the standard deviations of the fractions of samples in the collection of the 6 other non-centenarian (ie non-target or NT) groups, corresponding to healthy non-obese males, obese young males, diabetics with and without angiopathy, Alzheimer's and Parkinson's disease patients. The statistics *L_S_* is motivated by an analogy with the t-test.

A similar score that measures haplogroup enrichment for longevity is defined as: 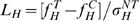



This measure is defined for each haplogroup H. *f_H_^T^* is the fraction of samples in the target (longevity) group (either centenarians or semi-supercentenarians) that belong to the haplogroup H, *f_S_^C^* is the fraction of samples in the control group (healthy non-obese males) that belong to the haplogroup H and *σ_H_^NT^* is the standard deviation of these fractions across the 6 non target groups.

We used the method of [13] to identify maximal bi-cliques. We define a biclique as a set of polymorphisms shared by at least 5 samples across all the data, independent of the haplogroups or phenotypes. The algorithm looks for combinations of sets of mutations (bi-cliques) that are shared by five or more individuals in the data, using a recursive algorithm which combines the sets onto bigger sets until all maximal sets are identified. The software for this algorithm along with a brief description is available at the website: http://genome.cs.iastate.edu/supertree/download/biclique. These bi-cliques define “patterns of mutation” which seem to be associated synergistically to longevity. The association of each pattern to longevity is estimated using an enrichment score *L_P_* for patterns. 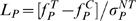



This statistic is defined for each pattern P. It represents the difference between the fraction of samples *f_P_^T^* that exhibit the pattern in the target longevity group (centenarians or semi-supercentenarians) minus the fraction of samples *f_P_^C^* that exhibit the same pattern in the control group, divided by the standard deviation *σ_P_^NT^* of these fractions over all the 6 non-target phenotypes.

The p-values for *L_S_, L_H_* and *L_P_* were calculated by comparing the experimental values to a null distribution created by computing their values in 1 million datasets obtained by a random permutation of phenotype labels. The fraction of values out of 1 million, that the enrichment score was greater than *L_S_, L_H_*, *L_P_* respectively, is the corresponding p-value. This permutation method for calculating p values is preferred because it avoids assumptions about the underlying distribution. We correct for the false discovery rate (FDR) using the method of Benjamini and Hochberg [14] which converts multiple hypothesis uncorrected p-values into corrected q-values [15].

Haplogroups associated with longevity were identified as those with a larger proportion of enriched patterns out of the total number of patterns associated with the haplogroup. The Fisher Exact test was used to examine the significance of the association between patterns and haplogroups. This test gives the exact probability of seeing a certain association between the two categorical variables by chance. We looked for associations between polymorphisms in the enriched patterns by identifying mutations which appear together in patterns and are simultaneously strongly associated with the longevity phenotypes. This was done by creating an agreement matrix *M = {m_ij_}* of size n x n, where n is the number of mtSNPs seen in the patterns. The entries *m_ij_* are defined so that they measure the beneficial effect of SNP *i* and SNP *j* if found in the same individual. Let *P* be the space of all patterns in the data and 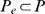 the subspace representing enriched patterns in semi-supercentenarians relative to healthy normals (q-value<0.05) and 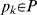 a pattern in this space; then the entries in the matrix *M* are:
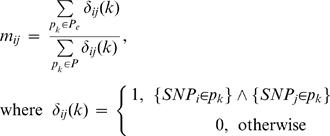



This score is similar to the pattern based feature selection defined in [16]. It is essentially the ratio between the numbers of times *SNP i* and *SNP j* appear together in the same enriched pattern and the total number of patterns that have both mutations. The agreement matrix is diagonal and symmetric. The diagonal entries represent the greatest possible correlation (because SNP i and SNP j are always found together in the same pattern if i = j). Setting the diagonal entries to 1 sets a threshold in the matrix for the greatest possible correlation between SNP i and SNP j. This is useful when we use the matrix to identify correlated cliques by thinking of it as a connectivity matrix for a graph where the nodes (labeled by the index i) are SNP locations and nodes i and j are considered linked by a bond if their correlation is greater than some threshold t. By successively lowering the value of the threshold from higher to lower values, one can identify the core set of SNPs that form a clique, which is a set of SNPs where each pair-wise correlation exceeds the threshold. Rows and columns of the matrix are then sorted (clustered) to place the clique SNPS in the top left corner. Color coding of the resulting matrix based on the entry values produces a heat map showing mtSNPs at different levels of enrichment.

To look for an association between the mtDNA polymorphisms and their haplogroups with nuclear mutations and pathways, we analyzed the 44 Japanese samples in the HapMap database [5]. Of these, 16 samples were classified by HapMap as belonging to the D clade using mtDNA markers. However, the SNP density in the HapMap database was insufficient for further sub-classification into D sub-haplogroups. Enrichment of mutations in the samples from the D clade with respect to the rest of the haplogroups was identified and its significance assessed using a chi-squared test.

It is known that population structure can introduce artificial correlations between SNPs and phenotypes. The classic example is SNPs in the lactase gene that spuriously correlates with the height phenotype. These spurious correlations can be easily understood by considering a simple example: imagine one homogeneous population that splits into two. Each of these populations evolves independently and develops a set of SNPs (markers) that clearly differentiate them. All these SNPs appeared as linked, because of the common ancestry of these populations. If, for any reason, one population has a different phenotype (height, disease, longevity) than the other, all these SNPs will correlate with the phenotype. However, this correlation does not mean that these SNPs are functional with respect to the phenotype. In fact, the association is merely due to the population structure of the data. It does not mean that the population does not have the distinguishing property as this is undisputable. What it means is that the association between the SNP and the phenotype is due to the appearance of the phenotype (for an unknown reason) after split from a common ancestor (ie. it is due to the phylogeny of haplogroups). Such correlations often introduce a high number of false positive spurious correlations in association studies.

Several methods [18]–[21] have been proposed to factor out associations due to population structure and identify real functional polymorphisms. For instance, structure association [19] classifies individuals into different clusters and looks for correlations within each cluster. Population genetic information is a combination of population structure and individual variations. The general idea in distinguishing mtDNA population structure polymorphisms from functional ones is to compare the eigenmodes of the actual SNP-SNP correlation matrix with those of a matrix of equal size but without population structure (generated using Normal Gaussian variables as described below), which, by construction, does not have any population structure[10], [18]–[21]. Population structure in the real mtDNA data shows up as large eigenvalues of the SNP-SNP correlation matrix. One can identify the polymorphisms that represent this population structure by looking at the eigenvectors corresponding to these large eigenvalues. The SNPs with large coefficients in these eigenvectors represent the polymorphisms which will be found on the tree representing the phylogeny of the samples in the data. If the data is projected onto these large eigenmode principal components, it will split into classes representing the phylogeny of their common ancestry. Thus, the eigenvectors corresponding to the set of large eigenmodes represents variation due to population structure and those corresponding to the set of small eigenmodes represent potentially real functional polymorphisms. If there is a strong correlation between the phenotype and the SNPs with large coefficients in the eigenvectors of large eigenmodes, then this represents population structure. Such correlations would then most probably originate in population structure related events, such as diet, climate, founder effects etc. Conversely, if the correlation is functional, there should be a strong correlation between the phenotype and the projection of the SNP matrix onto its lower eigenmodes. Such a correlation would be expected to be independent of population structure.

The decision on where to place the cutoff to separate “high” eigenmodes from “low” eigenmodes is made using the Tracy-Widom distribution (see [21] for a detailed description and references to the literature), which provides the basis for the null hypothesis. Consider a matrix M of dimension N_SNP_ x N_samples_ (where N_SNP_ and N_samples_ represent the number of SNPs and the number of samples in the original data). Assume that the entries M_ij_ are independent standard Normal Gaussian distributed variables for each SNP location. The product of M and its transpose M^T^ represents the correlation matrix of SNP-SNP correlations which would be obtained by chance. The distribution of eigenvalues of MM^T^ is the Tracy-Widom distribution which represents the null-probability to get a high eigenvalue by chance in data without population structure. We use a simple method (chosen to have a p-value<0.05) to find the cutoff x in eigenvalue to separate population structure from functional polymorphisms. We identify the highest k eigenvalues in the real data that represent more than a fraction f = 0.95 of the variation in the data as those representing population structure [21]. x is easily obtained by first finding k by solving: f = [Σ_i = 1_^k^ λ_i_]/N_SNP_ where the eigenvalues λ_i_ are sorted from highest to lowest value. The value of x is then obtained from: x = (λ_k_+λ_k+1_)/2.

## Results

**Figure 2:**
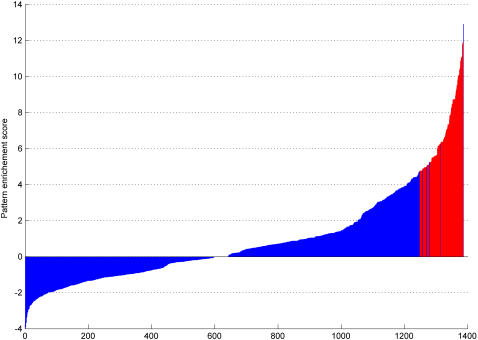
The significance scores for the 1386 maximal patterns of mutations in the data. The red region represents 124 patterns with q-value<0.05. The pattern enrichment score used here is L_P_.

**Table S2** shows the number of samples in each haplogroup in each of the 8 phenotypes. For statistical relevance, we have limited the haplogroup granularity to maintain at least 5 samples in each phenotype. Calculation of the *L_H_* score showed that only haplogroup D4a was enriched for longevity. Relaxing the p-value cutoff showed that the longevity signal is diffused among D4 haplogroups, becoming strongest in D4a. We found 1386 maximal patterns of mutations shared by at least 5 samples. For each pattern, we computed the *L_P_* score and its q-value as described above. We identified 124 “positive” patterns which were enriched in semi-supercentenarians compared to healthy Normals. We found no “deleterious” patterns which were depleted in semi-supercentenarians compared to healthy normals. The *L_P_* scores for all patterns are shown in **Figure 2** and the enriched patterns are those shown in red.

**Figure 3:**
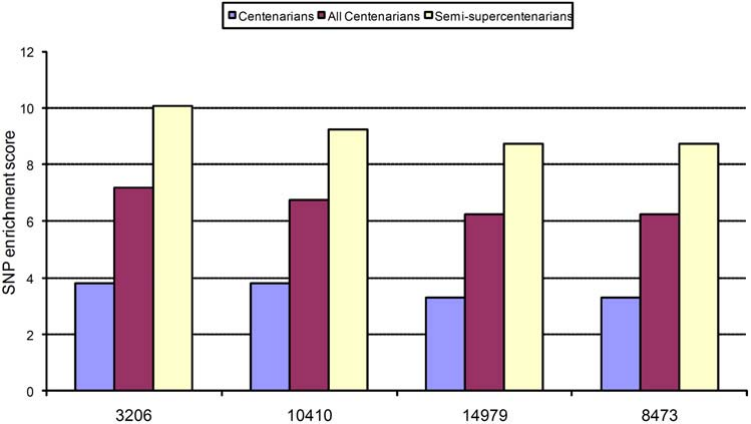
Significance score *L_S_* at q-value<0.05 for the enrichment of SNPs in the three data sets – 96 centenarians, 96+112 centenarians and semi supercentenarians, 112 semi-supercentenarians compared to healthy normals. 3206T, 14979C and 8473C are markers for D4a. SNP 10410C is enriched 80% in D4a vs its wild-type form 10410T.

**Figure 3** shows the mtSNPs enriched in the three datasets (centenarians, semi-supercentenarians and combined) compared to healthy normals using the score *L_S_* at q<0.05. Once again, we see a significant enrichment in several mtSNPs associated with the D4a haplogroup [6], [7], [12]. The top 4 enriched loci are 3206T, 8473C, 14979C and 10410C. The first three are defining markers for D4a. SNP 10410C is enriched (80% to 20%) in D4a compared to its wild-type form 10410T.

**Figure 4:**
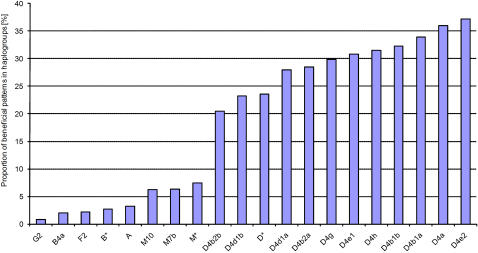
Proportion of beneficial patterns in haplogroups  = 100× (Number of positive patterns found in haplogroup)/(Total number of patterns found in haplogroup). In this analysis, we used the 124 positive patterns from Figure 2 with q-value<0.05.

The frequencies of the enriched patterns in various haplogroups are shown in **Figure 4**. The ordinate is defined as the proportion of beneficial patterns in the haplogroups  = 100× (Number of positive patterns found in haplogroup)/(Total number of patterns found in haplogroup). One of the haplogroups most enriched for longevity is again identified as D4a together with many haplogroups in the D4 clade followed by a smaller signal in other haplogroups outside the D clade. These results confirm our previous observation [17] that the longevity phenotype is enhanced for many of the D clade haplogroups.

**Figure 5:**
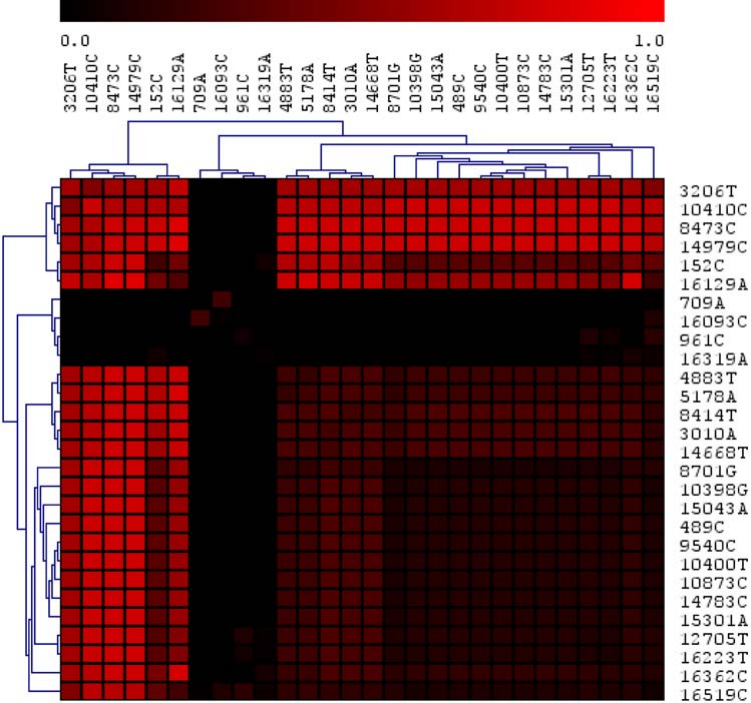
Heat map of the agreement matrix for SNPs across significant patterns. Note the presence of a group of 6 SNPs (upper left hand red box) strongly associated with longevity. These are: 3206T, 10410C, 8473C, 14979C, 152C and 16129A. The ones in bold are the same as in Figure 3 and are markers for D4a. The locations 152 and 16129 are known mutational hotspots in the hypervariable regions HVS-II and HVS-I respectively and hence their correlation with longevity cannot be considered significant.

The results of the agreement matrix analysis of mtSNPs in enriched patterns are shown in **Figure 5**. This heat map shows pairs of mtSNPs found in positive patterns across all haplogroups. Note the presence of a strong red block of SNPs corresponding to haplogroup D4a. The rest of the SNPs are enriched at lower values and could explain the presence of a weaker longevity signal among the rest of the D4 haplogroups.

**Table 1** shows a summary of the SNPs from the heat map. Again, we note that there are four strong SNPs (3206T, 8473C, 14979C and 10410C which are markers for D4a. There are also 5 non-synonymous mutations: 5178A, 8414T, 8701G, 10398G and 14979C. The first 3 polymorphisms are outside haplogroup D4a (specifically, they are defining mutations for D, D4, M resp.) while 14979C is D4a specific.

**Table 1:** List of most frequent mtSNPs from enhanced patterns (see Figure 5) in semi-supercentenarians vs. healthy normals.

SNP	Haplogrooup specificity	rCRS	Mutant	Gene	Sense
**152C**	**None**	**T**	**C**	**HVS-II**	
489C	M Clade, J	T	C	HVS-III	
709A	B5, G, M10, B4c, H13a	G	A	12S rRNA	
961C	None	T	C	12S rRNA	
3010A	D4 and many other groups	G	A	16S rRNA	
**3206T**	**D4a**	**C**	**T**	**16S rRNA**	
4883T	D	C	T	ND2	Syn
5178A	D	C	A	ND2	Nsyn
8414T	D4	C	T	ATP8	Nsyn
**8473C**	**D4a**	**T**	**C**	**ATP8**	**Syn**
8701G	M Clade	A	G	ATP6	Nsyn
9540C	M Clade	T	C	CO3	Syn
10398G	None	A	G	ND3	Nsyn
10400T	M Clade	C	T	ND3	Syn
**10410C**	**D4a**	**T**	**C**	**tRNA-Arg**	
10873C	M Clade	T	C	ND4	Syn
12705T	None	C	T	ND5	Syn
14668T	D4	C	T	ND6	Syn
14783C	M Clade	T	C	Cytb	Syn
**14979C**	**D4a**	**T**	**C**	**Cytb**	**Nsyn**
15043A	M Clade	G	A	Cytb	Syn
15301A	M Clade	G	A	Cytb	Syn
16093C	None	T	C	HVS-I	
**16129A**	**None**	**G**	**A**	**HVS-I**	
16223T	None	C	T	HVS-I	
16319A	None	G	A	HVS-I	
16362C	None	T	C	HVS-I	
16519C	None	T	C	HVS-I	

Column 2 shows the haplogroup specificity for the SNP. The next two columns show the nucleotide in rCRS and the mutation. The last two columns are for the corresponding gene and the ‘sense’ of the mutation – synonymous or nonsynonymous. SNPs shown in bold blue are the six SNPs in the top left corner of Figure 5, the ones most correlated with patterns enriched in the longevity group. Note that these are either non-specific or markers for haplogroup D4a. The locations 152 and 16129 are known mutational hotspots in the hypervariable regions HVS-II and HVS-I respectively and hence their correlation with longevity cannot be considered significant.

One way to identify the possible functional basis of the mtDNA enrichment of D4a in longevity is to look for autosomal polymorphisms that are also enriched in D4a over other haplogroups. If SNP data on autosomes for our samples were available, we could look such enrichments directly. However, in the absence of such data, we looked in the HapMap database [5] for polymorphisms on autosomes that were significantly enriched in D4a. Unfortunately, the SNP coverage in HapMap was too sparse to even identify the D4a haplogroup within the 44 Japanese samples in the D clade in the HapMap data. The best we could do was to see if these 44 D clade samples as a group had any significant autosomal enrichment. Such an analysis (of the HapMap D clade samples taken together as a single group) identified a number of enriched autosomal polymorphisms. The top 26 by p-value are shown in **Table 2** **.** One of these polymorphisms, on chr7:17152350, was within an exonic region and was simultaneously non-synonymous. Several others were in intronic, promoter and non-coding regions. In addition to the SNPs shown in **Table 2**, we found three other non-synonymous coding sites that were also enriched in D but at lower p values: chr3:122690866(H/R) in gene POLQ (DNA polymerase) with a p-value = 0.0004 and chr17:53939507(S/G), chr17:53940871(L/V) both in gene MTMR4 with p-values 0.0017 and 0.0024. MTMR4 is a dephosphorylation enzyme and exerts an effect on EGF (epidermal growth factor) receptor trafficking and degradation. The low density of SNPs in HapMap and the limited coverage of mtDNA polymorphisms limits our ability to do a more detailed study. The defining SNP 14979C for D4a was not measured in the HapMap project, precluding us from analyzing its possible correlation with autosomal SNPs.

**Table 2:** The top enhanced SNPs in the D clade in the Japanese samples in Hapmap [5].

#SNP rs#	SNP position	Gene(s)	Role	aa change	genotypes	p-value
rs12960296	chr18:49408898				GG[68.75%] GT[18.75%] TT[12.5%]	1.11E-05
rs17439885	chr18:49417508				AA[68.75%] AG[18.75%] GG[12.5%]	1.11E-05
rs5766424	chr22:43823070				AA[6.25%] AG[81.25%] GG[12.5%]	1.57E-05
rs3802082	chr7:17143421	AHR	Intron	-	AA[62.5%] AT[31.25%] TT[6.25%]	1.63E-05
rs13381188	chr18:49397848				CC[66.66%] CT[20.00%] TT[13.33%]	2.99E-05
rs4799386	chr18:31341066	C18orf37	Promoter	-	AA[56.25%] AC[43.75%] CC[0.0%]	3.63E-05
rs7791070	chr7:17174267				CC[6.25%] CT[37.5%] TT[56.25%]	4.08E-05
rs12232754	chr18:31309143	C18orf37	Intron	-	CC[56.25%] CT[43.75%] TT[0.0%]	4.74E-05
**rs2066853**	**chr7:17152350**	**AHR**	**exon**	**R/K**	**AA[12.5%] AG[31.25%] GG[56.25%]**	**4.95E-05**
rs10269143	chr7:17172223				AA[12.5%] AT[31.25%] TT[56.25%]	5.02E-05
rs10274243	chr7:17176936				AA[12.5%] AG[31.25%] GG[56.25%]	5.02E-05
rs7780687	chr7:17178958				AA[56.25%] AG[31.25%] GG[12.5%]	5.02E-05
rs17137566	chr7:17133761	AHR	Intron	-	CC[6.25%] CT[37.5%] TT[56.25%]	5.33E-05
rs3910440	chr12:29568570	ARG99	Intron	-	AA[18.75%] AG[6.25%] GG[75.0%]	5.79E-05
rs299454	chr12:29579764	ARG99	Intron	-	CC[18.75%] CT[6.25%] TT[75.0%]	5.79E-05
rs299467	chr12:29592557	ARG99	Intron	-	AA[75.0%] AG[6.25%] GG[18.75%]	5.79E-05
rs299470	chr12:29593452	ARG99	Intron	-	AA[75.0%] AG[6.25%] GG[18.75%]	5.79E-05
rs299479	chr12:29600357	ARG99	Intron	-	CC[75.0%] CT[6.25%] TT[18.75%]	5.79E-05
rs148898	chr12:29606383	ARG99	Intron	-	CC[75.0%] CG[6.25%] GG[18.75%]	5.79E-05
rs299487	chr12:29608184	ARG99	Intron	-	CC[75.0%] CT[6.25%] TT[18.75%]	5.79E-05
rs10169728	chr2:65917282	FLJ16124	Intron	-	AA[75.0%] AC[6.25%] CC[18.75%]	5.79E-05
rs733312	chr12:41827766				AA[81.25%] AG[18.75%] GG[0.0%]	5.81E-05
rs8096007	chr18:2199867				AA[75.0%] AG[18.75%] GG[6.25%]	8.55E-05
rs11188799	chr10:98250196	TLL2	Intron	-	CC[12.5%] CT[18.75%] TT[68.75%]	9.16E-05
rs9528775	chr13:63976625				CC[31.25%] CT[12.5%] TT[56.25%]	9.89E-05
rs7996275	chr13:64055128				CC[31.25%] CT[12.5%] TT[56.25%]	9.89E-05

We compare the D clade samples with the rest of the Japanese samples. Enrichment of the SNP was determined using a chi-squared test at 0.0001 significance level. The choice of mtSNPs in Hapmap was too sparse to resolve the D samples into subhaplogroups. We also cannot correlate SNP 14979C with autosomal SNPs because no D4a defining markers were available in the HapMap dataset.

We now apply Principal Component Spectral Analysis [10], [18]–[21] to the SNP-SNP covariance matrix to split the mtSNPs between those due to population structure (highest eigenmodes) and those with functional significance (the lowest eigenmodes). Using the cutoff value x = 0.95 for the Tracy-Widom distribution [21] we found that the top 40 eigenvalues correspond to population structure. Projecting the observed mtSNP matrix into the subspace orthogonal to that of these 40 eigenvalues and computing correlations, we find that the most correlated mtSNP now is 489C with r_P_ = 0.107 with p-value = 0.4 by the permutation test. This result suggests that there is no significant correlation between the individual genetic information and the phenotype.

If instead, we consider the sub-space of the 40 top eigenvalues, we find, in agreement with the previous analysis, that there are many mtSNPs that correlate with the phenotype. After correcting for multiple hypotheses (which indicated a cutoff abs(r)>0.15 for p-value<0.02) we found that the following 45 mtSNPs were significantly correlated with longevity: 103A, 194T, 199C, 489C, 1382C, 3010A, 3206T, 4048A, 4071T, 4164G, 4883T, 5178A, 5351G, 5460A, 6455T, 6680C, 7684C, 7853A, 8020A, 8251A, 8414T, 8473C, 8784G, 8964T, 9296T, 9824A, 10104T, 10345C, 10400T, 10410C, 12405T, 12811C, 13651G, 13708A, 14133G, 14605G, 14668T, 14783C, 14979C, 15043A, 15301A, 15524G, 16129A, 16297C, and 16362C. The mtSNPs undelined in this list were also identified in the analysis described in the previous section.

This analysis suggests that there is no functional mtSNPs significantly correlated with longevity. All significant correlations seem to be associated only with population structure (phylogenetic tree), suggesting that the significant longevity enrichment found in D4a (which is not in doubt) has causes other than mtDNA SNPs alone.

## Discussion

A strong finding of this paper, that is consistent across many different analysis techniques, is that in Japanese individuals are more likely to live longer if they belong to the D4a haplogroup. This result is also supported by previous studies [6], which found that the frequency of D4a in Japanese samples (*n* = 1312) was 7.39%. The overall frequency of 14979C (a marker of D4a) in our control samples was 8.44% (477 of 5651); 9.6% in Gifu (263 of 2748) [22], [23], 6.9% in Tokyo (103 of 1493) [unpublished data], and 7.8% in Gunma (111 of 1418) [unpublished data]. In our semi-supercentenarian samples, it was significantly enriched to 15.18%.

Our results suggest a possible scenario for the evolution of a longevity phenotype, which may develop by combinations of protective mutations. The signal for longevity in our data is strongest in D4a because it has the greatest number of protective mutations. Our spectral analysis suggests that the longevity phenotype must require protective mutations on nuclear chromosomes in addition to the defining mtDNA SNPs for D4a. Our rather limited association study on Japanese sequences in HapMap [5] identified a few coding SNPs associated with the D clade. The most enriched of these corresponds to the gene AHR [23] which is an Aryl hydrocarbon receptor and regulates several cytochrome P450 genes. It is not clear how these mutations affect longevity. Larger data sets with denser SNP coverage will be necessary to validate our results and generate hypothesis about possible mechanisms and pathways that contribute to longevity.

We attribute the reduction of the longevity effect seen in D4b2b [7] compared to D4a when moving from centenarians to semi-supercentenarians to the presence of 14979C (Cytb: Ile78Thr) in D4a. The hypothesis is that 5178A (ND2: Leu237Met) is associated with an overall protective effect for both groups, but the additional mutation at 14979C in D4a is correlated with a significant additional longevity effect in D4a.

A limitation of our study is the small sample size and lack of direct correlation with SNPs on nuclear chromosomes. We are currently trying to mitigate this problem by identifying nuclear SNPs associated with our samples on the complexes formed by proteins encoded by mtDNA. Adaptive selection of mitochondrial complex I subunits during primate evolution has been reported to support the concept of co-evolution of mtDNA-encoded and nDNA-encoded subunits of the OXPHOS complexes [25]. Experiments with xenocybrids carrying human nuclear genome and various primate mitochondrial genome indicate that the mismatches between mtDNA and nDNA gene products within the OXPHOS complexes result in decreased enzyme activities and/or increased production of reactive oxygen species from the mitochondria [26]–[28]. The finding that subhaplogroup D4a is one of the most frequent ones in Japanese suggest that this mitochondrial subhaplogroup may be compatible with the major variants of the nDNA-encoded subunits of the OXPHOS complexes. Over a longer term, we plan to apply the techniques discussed in this paper to more extensive genome wide association studies using SNP chips to find pathways correlated with complex diseases such as type 2 diabetes, nephropathy, myocardial infarction etc. In conclusion, the nuclear-mitochondrial interaction model for longevity may be an attractive working hypothesis to be examined by resequencing projects targeting Aisan- or Japanese-specific nuclear genome variations. The resequencing projects focusing on mitochondrial genome in Japanese semi-supercentenarian reported here is a landmark for the start of investigation on longevity-associated nuclear genome polymorphisms.

Many mutational hotspots are known to exist in mtDNA (see section ‘Estimation of Mutation rates and coalescence times: some caveats' in [29] for details). These are mainly concentrated in the hypervariable regions of the mtDNA control region. In particular, the locations 16129, 152 and 489 etc, which appear in **Table 1** and **Figure 5** are mutational hotspots in the hypervariable regions HVS-I, HVS-II and HVS-III respectively. They are commonly found in several unrelated haplogroups. Similarly, the locus 3010 is also a mutational hotspot found in many disparate haplogroups. Because of this, it is difficult to convincingly demonstrate and/or validate any possible association of these hotspot polymorphisms with longevity. It is difficult to say whether their appearance in our analysis is due to chance or reflects some deeper epigenetic effect in association with other polymorphisms on autosomes.

The enriched loci identified in this study are 3206T, 8473C, 14979C and 10410C of which the first three are defining markers for D4a and the fourth (10410C) is enriched (80% to 20%) in D4a compared to its wild-type form 10410T. This polymorphism is a known identifying marker for subhaplogroup D4a1 [30]. Its identification in our analysis may reflect a further refinement of the association of longevity within D4a. However, our sample size is inadequate to pursue this further in the present study.

There are two ways in which any group of polymorphisms might associate with a phenotype. The most direct is if their presence alone confers the phenotype. An indirect way is if the polymorphisms are merely associated (correlated) with the phenotype because of population structure. Our spectral analysis suggests that the latter association is what is actually realized in the data. This does not mean that the association between D4a and longevity is spurious – only that the correlation between the defining SNPs for D4a and longevity is spurious. The longevity phenotype appears to correlate with D4a because it appeared due to factors other than the defining SNPs of D4a. Whereas there are many other sources of the association (diet, lifestyle etc), one possibility is that the D4a haplogroup is enriched in autosomal SNPs that confer the longevity phenotype. Whereas our paper raises this as a strong possibility, our efforts to identify such autosomal correlations in the HapMap dataset were unsuccessful because of the limited sample size of the D haplogroup and the limited SNP coverage in the HapMap study. The hypothesis of association between D4a and longevity due to autosomal polymorphisms can be tested by a study involving more individuals in the D haplogroup in Japan and elsewhere. It is particularly important in this context to include D4a individuals outside Japan to rule out associations due to diet and lifestyle.

## Supporting Information

Data S1 mtDNA sequences of 112 semi-supercentenarians in FASTA format.
(1.87 MB TXT)

Table S1 Table of mtSNPs detected in each individual.
(2.75 MB XLS)

Table S2 This table shows the number of samples in each robust haplogroup in each of the 8 phenotypes. Ambiguous sequences, which could not be classified based on both our clustering system and standard markers in the literature are labelled with *. Thus, M* labels those samples which can be classified into the M clade but not further and so on.
(0.09 MB DOC)
