# Supplementary material for: Mitochondrial DNA Haplogroup D4a Is a Marker for Extreme Longevity in Japan
Source: PLoS One. 2008 Jun 11;3(6):e2421. doi: 10.1371/journal.pone.0002421 (PMC2408726; doi:10.1371/journal.pone.0002421)
Supplement: Table S2 — This table shows the number of samples in each robust haplogroup in each of the 8 phenotypes. Ambiguous sequences, which could not be classified based on both our clustering system and standard markers in the literature are labelled with *. Thus, M* labels those samples which can be classified into the M clade but not further and so on. (0.09 MB DOC) [file pone.0002421.s003.doc]

|  | diabetic patients with severe angiopathy | type-2 diabetes mellitus patients | healthy non-obese young males | obese young males | patients with Alzheimer’s disease | patients with Parkinson’s disease | centenarians | semi-super-centenarians |
| --- | --- | --- | --- | --- | --- | --- | --- | --- |
| A | 8 | 9 | 4 | 9 | 5 | 5 | 8 | 7 |
| B4a | 3 | 0 | 3 | 3 | 1 | 3 | 0 | 6 |
| B4b | 6 | 1 | 3 | 3 | 1 | 2 | 2 | 2 |
| B4c | 3 | 0 | 2 | 5 | 5 | 3 | 2 | 4 |
| B5 | 6 | 3 | 1 | 5 | 4 | 2 | 4 | 4 |
| C | 0 | 0 | 1 | 0 | 0 | 0 | 1 | 0 |
| D4a | 7 | 6 | 4 | 6 | 5 | 5 | 8 | 17 |
| D4b1a | 1 | 1 | 0 | 0 | 1 | 1 | 1 | 2 |
| D4b1b | 2 | 0 | 4 | 1 | 2 | 3 | 1 | 1 |
| D4b2a | 3 | 1 | 6 | 1 | 2 | 2 | 6 | 2 |
| D4b2b | 3 | 7 | 2 | 4 | 9 | 4 | 11 | 6 |
| D4d1a | 0 | 0 | 2 | 0 | 1 | 3 | 4 | 0 |
| D4d1b | 0 | 1 | 0 | 4 | 2 | 0 | 1 | 0 |
| D4e1 | 1 | 3 | 2 | 1 | 0 | 0 | 1 | 6 |
| D4e2 | 7 | 2 | 3 | 3 | 3 | 4 | 2 | 1 |
| D4g | 1 | 2 | 5 | 5 | 1 | 1 | 1 | 2 |
| D4h | 0 | 2 | 0 | 2 | 2 | 1 | 0 | 1 |
| D5a | 1 | 3 | 0 | 2 | 3 | 3 | 4 | 1 |
| D5b | 1 | 2 | 1 | 2 | 1 | 2 | 3 | 1 |
| F1 | 1 | 3 | 5 | 0 | 1 | 1 | 2 | 5 |
| F2 | 3 | 3 | 4 | 3 | 6 | 2 | 2 | 3 |
| G1 | 6 | 4 | 1 | 3 | 4 | 6 | 1 | 3 |
| G2 | 6 | 2 | 4 | 4 | 8 | 4 | 5 | 4 |
| M10 | 0 | 2 | 1 | 1 | 0 | 0 | 0 | 2 |
| M7a | 1 | 7 | 10 | 4 | 6 | 10 | 7 | 8 |
| M7b | 2 | 3 | 1 | 7 | 4 | 5 | 7 | 7 |
| M8 | 0 | 5 | 2 | 0 | 1 | 2 | 0 | 0 |
| M9 | 2 | 2 | 4 | 1 | 0 | 2 | 0 | 3 |
| N9a | 9 | 3 | 4 | 5 | 5 | 6 | 2 | 5 |
| N9b | 2 | 4 | 3 | 5 | 4 | 0 | 0 | 1 |
| Z | 2 | 1 | 0 | 1 | 0 | 3 | 1 | 0 |
| B* | 1 | 2 | 1 | 1 | 2 | 1 | 4 | 0 |
| N* | 0 | 2 | 3 | 0 | 2 | 0 | 0 | 0 |
| M* | 2 | 1 | 5 | 1 | 1 | 1 | 1 | 1 |
| D* | 6 | 9 | 5 | 4 | 4 | 9 | 4 | 7 |
